# CX3CL1/CX3CR1 axis in liver disease: context-dependent roles and balance

**DOI:** 10.3389/fimmu.2026.1763348

**Published:** 2026-02-04

**Authors:** Jing Liu, Zhen Guo, Xin Zheng

**Affiliations:** 1 Department of Infectious Diseases, Union Hospital, Tongji Medical College, Huazhong University of Science and Technology, Wuhan, China; 2 National 111” Center for Cellular Regulation and Molecular Pharmaceutics, School of Life and Health Sciences, Hubei University of Technology, Wuhan, China

**Keywords:** CX3CR1, fractalkine (CX3CL1), immune regulation, liver diseases, targeted therapy

## Abstract

This review provides a systematic and critical examination of the multifaceted roles of the CX3CL1/CX3CR1 axis in liver diseases. We emphasize its context-dependent duality—exhibiting both pro- and anti-inflammatory, pro- and anti-fibrotic, and pro- and anti-tumor functions across different etiologies. Moving beyond a binary good or bad” paradigm, we propose a contextual signaling model that integrates cellular source, microenvironmental cues, and intersecting pathways to explain its divergent roles. We synthesize recent advances in its involvement in NAFLD/NASH, viral hepatitis, autoimmune hepatitis, schistosomiasis, liver fibrosis and hepatocellular carcinoma. The review critically evaluates the axis’s potential as a biomarker, discusses methodological advances and limitations in human studies, and analyzes therapeutic strategies with a focus on translational challenges. We conclude with a forward-looking perspective on precision medicine approaches targeting this axis.

## 1 Introduction

The liver is a central organ for metabolism, detoxification, and immune regulation. Chronic liver injury, driven by factors such as viral infections, metabolic dysfunction, or toxins, triggers complex interactions between hepatocytes, immune cells, and stromal cells, leading to inflammation, fibrosis, and cancer. The fractalkine (CX3CL1)/CX3CR1 axis, a chemokine system with dual adhesive and migratory functions, has emerged as a key player in these processes. CX3CL1 exists in both soluble and membrane-bound forms, enabling it to act as a chemoattractant for CX3CR1-expressing immune cells (e.g., monocytes, NK cells, and T cells) or as an adhesion molecule. Early studies highlighted its role in liver inflammation and fibrosis, but recent research has revealed a profound context-dependent duality in its functions. This review aims to critically synthesize the current understanding of the CX3CL1/CX3CR1 axis across the spectrum of chronic liver disease—from the initial stages of persistent inflammation and subsequent fibrogenesis to the ultimate development of hepatocellular carcinoma. By integrating recent mechanistic advances within this progression framework, we will propose conceptual models to explain its functional plasticity and guide future therapeutic development.

## 2 Overview of the CX3CL1/CX3CR1 axis in liver pathophysiology

The CX3CL1/CX3CR1 axis plays a crucial role in liver pathophysiology. CX3CL1, also known as fractalkine, and its receptor CX3CR1 are involved in various processes related to liver diseases. In metabolic-associated fatty liver disease, increased CX3CL1 in hepatic stellate cells (HSCs) inhibited Kupffer cell autophagy and promoted cell apoptosis by interacting with CX3CR1. The results innovatively expand the axis’s function beyond chemotaxis by uncovering a novel intracellular pathway that regulates Kupffer cell autophagy and survival (1). However, this discovery is confined to a model of chronic intermittent hypoxia, creating an information silo” and leaving its role in common conditions like metabolic steatohepatitis unclear.

The previous studies established the clinical relevance of the CX3CL1/CX3CR1 axis in specific human liver diseases—primary biliary cirrhosis (2) and chronic hepatitis C (3)—and in identifying activated HSCs as a critical cellular source of the ligand in chronic inflammation (4). Unfortunately, correlative evidence showed expression patterns or genetic associations without demonstrating direct causality in disease pathogenesis. This stems primarily from the methodological constraints of their time, lacking advanced tools for cell-specific genetic manipulation within complex organ systems to move beyond observational and *in vitro* findings.

Early clinical observations also identified CX3CL1 as a key chemokine in primary biliary cirrhosis, where the CX3CL1-CX3CR1 axis was implicated in aggravating inflammation (5). Subsequent research confirmed that CX3CR1 played a protective role against liver fibrosis by controlling infiltrating hepatic monocytes (6). These results offered valuable early insights but were limited by their reliance on global genetic knockout models. While this approach is powerful, it cannot distinguish the effects in different myeloid cell subsets (e.g., Kupffer cells vs. infiltrating monocytes) or at different disease stages. Consequently, the precise cellular mechanisms of protection vs. aggravation remain partially obscured. Modern techniques like intravital imaging are now revealing even more complex systemic interactions, such as splenic classical monocytes remotely modifying hepatic CX3CR1+ cell motility to exacerbate fibrosis via the spleen-liver axis (7). This finding powerfully illustrates that the axis’s pathophysiological impact can transcend the local liver environment, adding a layer of systemic complexity that earlier studies could not capture (**Table 1**).

**Table 1:** The role of the CX3CL1/CX3CR1 axis in liver pathophysiology and diseases.

Study model	Species	CX3CL1 source cell	CX3CR1^+^ effector cell	Major pathway	Outcome
Viral Hepatitis (HBV/HCV) (3, 18)	Human	Hepatocytes, Hepatic stellate cells	Infiltrating monocytes/macrophages, T cells	NF-κB	In chronic HCV infection, CX3CR1 is directly involved in promoting liver fibrosis. Conversely, in HBV infection, lower plasma CX3CL1 levels correlate with advanced fibrosis stage, suggesting a potential exhaustion of a protective pathway or increased ligand consumption at injury sites.
Primary biliary cholangitis (PBC) (2, 5)	Human	Biliary epithelial cells (BECs)	CD3^+^ T cells, CD68^+^ macrophages	TLR3/4, STAT	In PBC, senescent cholangiocytes upregulate CX3CL1. This recruits CX3CR1^+^ monocytes and T cells to periductal areas, driving biliary-specific inflammation and injury.
Autoimmune hepatitis model (Con A-induced) (27)	Mouse	Not specified	Monocytes, T cells (CD4^+^, CD8^+^), NKT cells	TLR4/MyD88/NF-κB	The CX3CL1/CX3CR1 axis is a downstream effector of pathogenic NF-κB signaling in immune-mediated hepatitis. It promotes the infiltration of CD4^+^, CD8^+^ T, and NKT cells, exacerbating injury. Pharmacological inhibition of the axis (via Protect D1) by suppressing NF-κB activation reduces immune cell recruitment and protects against liver damage, highlighting its pathogenic role and therapeutic target potential in this context.
Schistosomiasis animal model (30, 40)	Mouse	Granuloma outer layer cells	Infiltrating macrophages	STAT6, PPAR-γ	In schistosome infection, CX3CR1 deletion alleviates hepatic granuloma and injury by promoting M2 macrophage polarization and enhancing the Th2 immune response.
NASH mouse model (9, 43)	Mouse	Hepatocytes, Hepatic stellate cells, Intestinal cells	Macrophages	NF-κB, p38 MAPK, TLR4	CX3CR1 deletion exacerbates disease by promoting pro-inflammatory M1 macrophage polarization, whereas CX3CL1 overexpression demonstrates a protective effect, reversing insulin resistance, inflammation, and fibrosis. CX3CR1 deficiency disrupts intestinal barrier integrity, leading to increased bacterial translocation, hepatic TLR4 activation, and exacerbated steatohepatitis. CX3CR1 acts as a gatekeeper for intestinal homeostasis, thereby limiting NASH progression.
CCl_4_-induced liver Injury model (28)	Mouse	Hepatocytes, Hepatic stellate cells	Kupffer cells	AKT, ERK	CX3CR1 deletion aggravates injury and fibrosis by enhancing the pro-inflammatory phenotype of Kupffer cells. In contrast, CX3CL1 treatment induces an anti-inflammatory profile in Kupffer cells and directly inhibits HSC activation.
Liver fibrosis model (6)	Human/Mouse	Hepatocytes, hepatic stellate cells	Infiltrating monocytes/macrophages	Not specified	CX3CR1 signaling limits liver fibrosis by enhancing monocyte/macrophage survival (via Bcl-2), suppressing their M1 polarization, and downregulating TIMP-1 in hepatic stellate cells, thereby modulating inflammatory responses and extracellular matrix metabolism.
HCC model (11, 12)	Human/Mouse	Hepatocellular carcinoma cells, Hepatic stellate cells	NK cells, macrophages	STAT3	Tumor Suppressive Role: miR-561-5p promotes HCC growth by suppressing CX3CL1, reducing the infiltration and cytotoxicity of CX3CR1^+^ NK cells. Tumor Promotive Role: aHSC-derived retinoids induce Arg1 in CX3CR1^+^ macrophages, which suppresses CD8^+^ T-cell proliferation and promotes HCC. CX3CR1 knockout or ADH3 inhibition attenuates tumorigenesis.

## 3 Current understanding of CX3CL1/CX3CR1 in liver disease mechanisms

In immune-mediated hepatitis, Cx3cr1 deficiency exacerbates liver injury, suggesting a protective, anti-inflammatory role. This is supported by data showing that deficiency leads to heightened Nf-κB activation and pro-inflammatory cytokine production in macrophages and T cells (8). However, this protective effect stands in stark contrast to its role in metabolic disease. In non-alcoholic steatohepatitis (NASH), the same Cx3cr1 deficiency promotes inflammatory monocyte infiltration and a shift towards M1-dominant macrophages, worsening steatohepatitis (9). This paradox underscores a fundamental principle: the net effect of CX3CL1/CX3CR1 signaling is not intrinsic but is dictated by the disease microenvironment. The NASH microenvironment itself is characterized by dynamic shifts in the composition of resident and recruited macrophages that critically influence tissue remodeling (10). The mechanistic basis for this divergence may lie in the distinct immune drivers of each condition: in hepatitis, the axis may temper excessive adaptive immune responses (e.g., T cell and macrophage activation via NF-κB), whereas in NASH, it appears crucial for maintaining myeloid cell homeostasis, possibly by regulating macrophage migration and M1/M2 balance within the metabolically stressed liver.

The axis’s role in cancer further illustrates its complexity and potential for dual anti-tumor and pro-tumor functions, often building upon the inflammatory and fibrogenic niches it helped shape. As discussed in preceding sections, in chronic viral hepatitis or NASH, the axis participates in shaping the long-term inflammatory and fibrogenic microenvironment. Within this primed tissue context, its function can be repurposed to facilitate oncogenesis. On one hand, the miR-561-5p/CX3CL1 axis in hepatocellular carcinoma (HCC) influences the infiltration of CX3CR1+ natural killer cells, which play a role in anti-tumour immunity (11). Conversely, a pro-tumor role is evidenced by studies showing that CX3CR1+ macrophages interact with HSCs to suppress CD8+ T-cells and promote HCC progression (12). Recent work adds intricate layers to this pro-tumor narrative. For instance, direct platelet-tumor cell interaction can activate a TLR4/ADAM10/CX3CL1 axis to aggravate HCC metastasis (13). Paradoxically, another study found that platelets recruited via CX3CL1-CX3CR1 can induce tumor cell apoptosis (14), highlighting that even within the tumor niche, the role of a single cellular player (platelets) can be contradictory depending on other signals. Furthermore, the axis facilitates distant organ metastasis, as evidenced by bone marrow endothelial cell-derived, ADAM17-regulated CX3CL1 promoting HCC spinal metastasis (15). This underscores that the axis’s impact in oncology extends beyond the primary tumor to mediate devastating distal complications. The functional outcome likely depends on which CX3CR1+ immune cell subset is predominantly recruited—a factor influenced by the cellular source of CX3CL1 (e.g., hepatocytes vs. HSCs vs. endothelial cells) and other local signals. The discovery of specialized immune cell networks in the liver, such as distinct portals containing interconnected networks of CX3CR1+ macrophages and dendritic cells (16), and neuroprotective liver portal area macrophages that actively suppress inflammation (17), provides a sophisticated anatomical and functional substrate for this context-dependent signaling. These findings elevate our understanding from a simple ligand-receptor pair to a component.

In summary, the current understanding necessitates a model-specific and cell-specific interpretation of the CX3CL1/CX3CR1 axis. The field is moving away from the question Is the axis good or bad?” towards the more critical questions of Under what conditions, and through which cellular circuits, does it drive or suppress disease?” Resolving this requires integrated analysis across models and a focus on the human disease context. Accordingly, we propose a contextual signaling model wherein the functional outcome is determined by: (1) the cellular source of CX3CL1 (hepatocytes, HSCs, endothelial cells), (2) the recruited CX3CR1+ subset (monocytes, macrophages, NK cells, T cells), and (3) cross-talk with key pathways such as NF-κB, TLRs, metabolic sensors, and miRNAs. This model provides a framework for understanding duality and guiding therapeutic strategies.

## 4 Clinical and prognostic significance of the CX3CL1/CX3CR1 axis

While studies link the CX3CL1/CX3CR1 axis to various liver diseases, a critical synthesis reveals significant gaps in distinguishing causal drivers from passive correlates. The observed associations are compelling but often lack mechanistic depth in human contexts.

For instance, in chronic Hepatitis B (HBV), patients with severe fibrosis or cirrhosis exhibit significantly lower plasma CX3CL1 concentrations compared to those with mild disease (18). Similarly, serum fractalkine levels are positively associated with the clinical severity of liver cirrhosis (19), and its levels correlate with the severity of radiation-induced liver injury (20). While these correlations strengthen the link between the axis and disease burden, they fall short of proving utility as dynamic predictive or monitoring biomarkers. Crucially, they cannot distinguish whether altered CX3CL1 is a cause, a consequence, or an epiphenomenon of the disease state. Advanced computational models, such as the CX3CR1-associated gene signature for HCV cirrhosis prognosis (21) or the machine-learning identification of a NASH-macrophage” population (22), represent sophisticated correlations. However, they are inherently constrained by the biases and heterogeneity of their source datasets (e.g., limited sample size, specific patient subgroups). A key methodological limitation is overfitting, where models perform well on initial data but may fail in independent, diverse cohorts. Their current value is hypothesis-generating; robust external and prospective validation is the mandatory next step to assess true predictive power and generalizability before clinical application can be considered.

The definitive role of the axis in experimental hepatopulmonary syndrome (HPS) (23) and postoperative HCC recurrence (24) are clear examples of the causal inferences coming from controlled experimental settings. However, these mechanistic insights have yet to be translated into human epidemiology. The association of CX3CR1+ T-cells with cardiometabolic disease in people with HIV (25) is an important observation, but its evidence grade remains low due to confounding factors inherent to the HIV population and the observational study design. The field is over-reliant on associative human data and there is a paucity of interventional or genetic evidence (e.g. Mendelian randomization studies in humans). For most diseases, we cannot conclude that the axis is a causal driver in human populations. Its current epidemiological value is therefore primarily as a research marker, not a validated clinical tool. Studies designed to test causality and predictive utility in humans directly are needed to fill this translational gap.

## 5 Pathophysiological mechanisms of the CX3CL1/CX3CR1 axis in liver diseases

Chronic inflammation and the resultant fibrotic scarring establish the fundamental pathological soil for HCC. The CX3CL1/CX3CR1 axis exhibits a critical yet paradoxical duality across this pathogenic continuum, functioning as either a driver of injury or a protective mechanism depending on the specific disease stage and cellular context. In metabolic and immune-mediated conditions such as NASH and ConA-induced hepatitis, it acts as a pathogenic promoter (26, 27). In stark contrast, during CCl4-induced fibrosis, the axis transduces anti-inflammatory and anti-fibrotic signals, wherein Cx3cr1 deficiency paradoxically exacerbates injury (28). This protective effect may involve the modulation of intrahepatic macrophage polarization and chemotaxis, as suggested by studies on traditional formulas that act through Ccl2 and Cx3cr1 pathways (29). The protective role is not universal across fibrotic models, as deficiency can attenuate granuloma formation in schistosomiasis by enhancing M2 polarization (30), and splenic monocytes can exacerbate fibrosis by modifying hepatic Cx3cr1+ cell motility (7). This model-specificity is a major confounding factor in interpreting the axis’s role in fibrosis.

New therapeutic strategies continue to emerge from dissecting this network. For example, inducing HSC ferroptosis via the HIC1/CX3CL1/GPX4 axis presents a novel anti-fibrotic mechanism (31). In hepatic ischemia-reperfusion (I/R) injury, the paradox is also evident: blocking Cx3cl1 attenuates platelet and leukocyte recruitment, mitigating injury (32), while Cx3cr1 deficiency can promote resolution by regulating macrophage homeostasis (33). These seemingly contradictory findings in the same injury model may reflect differential effects on specific cell populations or injury phases, again emphasizing the need for cellular and temporal precision in mechanistic understanding.

**Figure 1:**
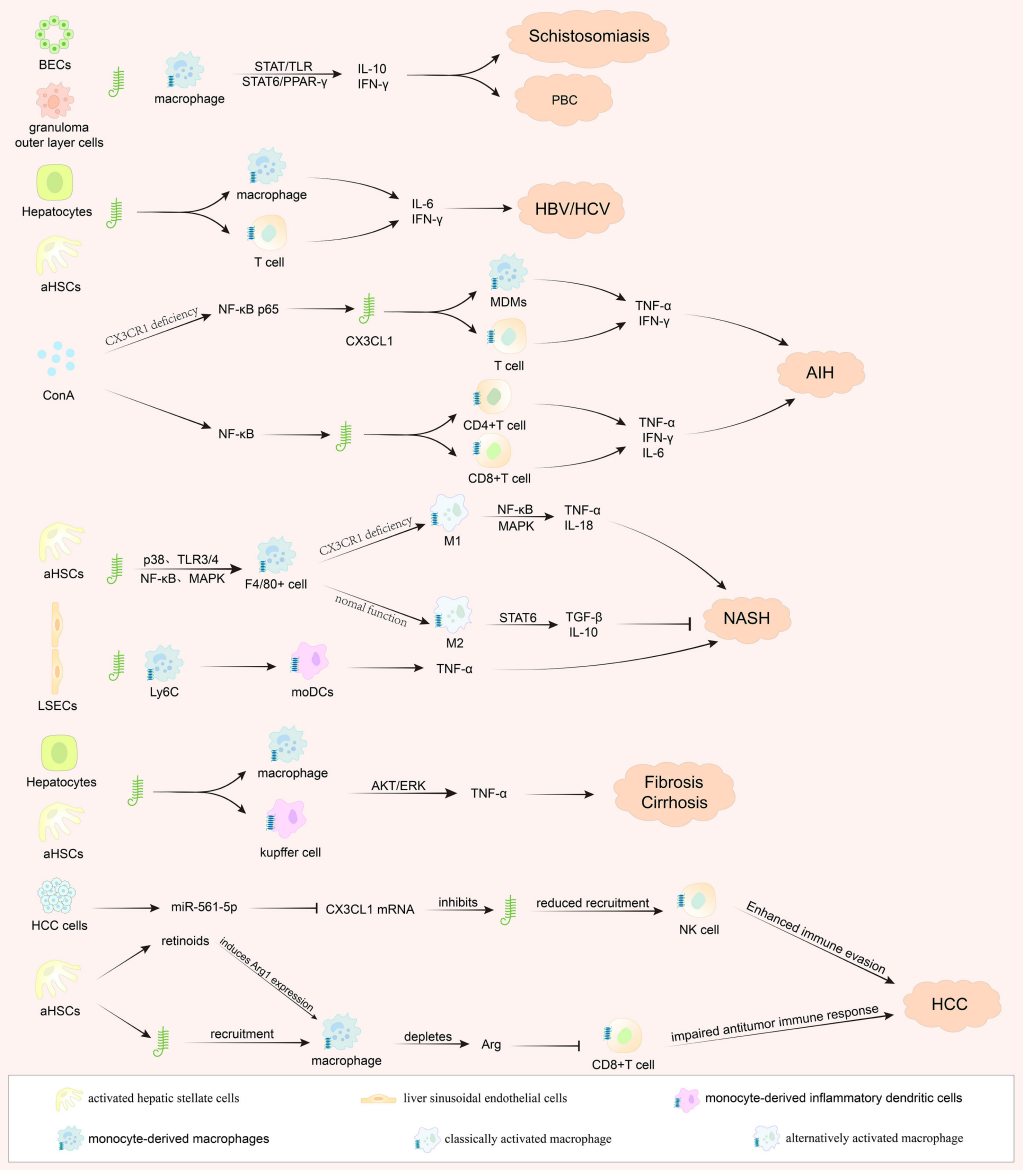
The CX3CL1/CX3CR1 axis orchestrates hepatic inflammation, fibrosis, and hepatocarcinogenesis. Schematic depicting the central role of the CX3CL1/CX3CR1 chemokine axis in driving disease progression from chronic liver injury to hepatocellular carcinoma (HCC). Upon sustained exposure to pathogen- or damage-associated molecular patterns (PAMPs/DAMPs) across various etiologies, resident liver cells—including liver sinusoidal endothelial cells (LSECs), activated hepatic stellate cells (aHSCs), biliary epithelial cells, and hepatocytes—upregulate the expression of CX3CL1. This promotes the recruitment of CX3CR1-expressing immune cells such as monocyte-derived macrophages (MDMs), Kupffer cells, and T cell subsets (CD4^+^ and CD8^+^). Once activated, these infiltrating and resident cells release pro-inflammatory cytokines (e.g., TNF-α, IFN-γ, IL-6, IL-17) and engage key signaling pathways (e.g., NF-κB, MAPK, STAT4), collectively perpetuating hepatic inflammation, activating aHSCs, and accelerating extracellular matrix deposition, thereby advancing fibrosis and cirrhosis. Within this established fibrotic and chronically inflamed microenvironment, persistent immune dysregulation and parenchymal injury foster a tumor-promoting niche, ultimately facilitating the development of HCC.

The axis’s regulatory network is complex. IFN-γ fine-tunes CX3CL1 availability via a KSRP/miR-27b-mediated mechanism (34), and the axis is co-activated with NF-κB and the NLRP3 inflammasome in autoimmune injury (27). Its impact extends beyond the liver: suppressed CX3CL1/CX3CR1 signaling in the brain exacerbates neuroinflammation in hepatic encephalopathy (35). Pharmacological studies show that this neuroinflammatory component can be targeted, as drugs can ameliorate hepatic encephalopathy by modulating C/EBP-β and CX3CL1/Fractalkine (36), and rifaximin can prevent cerebellar immune cell infiltration (37). These findings solidify the concept of a liver-brain axis mediated in part by CX3CL1/CX3CR1, revealing a potential therapeutic target for neurological complications of liver disease (**Figure 1**).

## 6 Diagnostic techniques for liver diseases involving the CX3CL1/CX3CR1 axis

The CX3CL1/CX3CR1 axis is emerging as a promising multi-faceted biomarker candidate across a spectrum of liver diseases, with its utility varying by disease context and the specific clinical need. In pediatric autoimmune hepatitis, proteomic analysis links CX3CL1 to an inflammatory network, but this association, derived from a small, specific cohort, cannot confirm its pathogenic role or diagnostic utility (38). Similarly, identifying CX3CR1 as a marker for NASH-macrophages” via single-cell sequencing is a powerful observation. However, it is constrained by the technical and biological heterogeneity of sequenced samples and does not establish whether CX3CR1 expression is a cause or consequence of disease (22). The axis demonstrates particular prognostic strength in hepatocellular carcinoma, where the infiltration of CX3CR1+ MDSCs and the assessment of CX3CR1-associated gene signatures are consistently linked to poorer patient outcomes, offering a potential tool for risk stratification in HCV-induced cirrhosis and HCC (13, 21, 39). However, a critical methodological limitation across these studies is the lack of prospective validation in independent, large-scale cohorts to assess generalizability beyond the initial discovery population.

Methodological advances, while sophisticated, have not yet bridged the gap to proving causality. Integrated techniques in schistosomiasis research correlate hepatic CX3CL1/CX3CR1 with serum fibrosis markers (40), and multi-platform approaches in HCC link plasma CX3CL1 to MDSC recruitment (39). These methods provide valuable spatial and correlative data. However, they remain observational. They cannot demonstrate that modulating the axis alters disease course—a key requirement for validating a therapeutic target or a dynamic biomarker. The root limitation is that these human and ex vivo studies are not interventional. Translating these technical findings into causal evidence requires functional validation in experimental models that can test the necessity and sufficiency of the axis, followed by human studies with interventions targeting this pathway.

Despite this promise, a critical synthesis identifies key challenges for future development. The field currently lacks standardized and validated assays for measuring soluble CX3CL1 or quantifying CX3CR1+ cell populations in clinical practice. Future research must prioritize the technical validation of these detection methods across different liver diseases and establish clear, context-specific cutoff values for clinical interpretation. Furthermore, large-scale longitudinal studies are needed to determine whether CX3CL1/CX3CR1 dynamics can reliably monitor treatment response or disease recurrence. Ultimately, unlocking the full diagnostic and prognostic potential of this axis depends on moving from associative findings to the creation of integrated models that combine CX3CL1/CX3CR1 metrics with other clinical and molecular parameters.

## 7 Therapeutic strategies targeting the CX3CL1/CX3CR1 axis in liver diseases

The transition from mechanistic understanding to therapeutic intervention for the CX3CL1/CX3CR1 axis necessitates a precision medicine approach, dictated by its context-dependent roles across liver diseases. This fundamental duality, observed across the disease continuum from inflammation to cancer, defines the core challenge: developing agents that can selectively inhibit pathogenic signaling without disrupting homeostatic or protective functions in other cellular compartments or disease stages. In HCC, where the axis facilitates an immunosuppressive microenvironment by recruiting CX3CR1+ MDSCs, antagonism is a validated strategy. Preclinical support for this includes the use of neutralizing antibodies to block CX3CL1, thereby impeding MDSCs recruitment and tumor progression (13, 39). Rational combination therapies, such as coupling axis inhibition with immune checkpoint blockade, are promising (12, 39). This approach can be augmented by strategies that broadly inhibit protumorigenic macrophage recruitment, such as blocking CacyBP, which has been shown to improve anti-PD-1 therapy efficacy in HCC (41). Conversely, in NASH, evidence suggests a protective role, where CX3CL1/CX3CR1 signaling can mitigate disease, indicating a potential for agonist-based therapies (9). This fundamental duality defines the core challenge: developing agents that can selectively inhibit pathogenic signaling without disrupting homeostatic or protective functions in other cellular compartments.

Translating these insights into clinical applications faces significant challenges but is guided by clear mechanistic opportunities. A major hurdle is the current absence of clinical trials directly targeting this axis in liver diseases, highlighting a critical translational gap. The primary challenge lies in developing agents that can selectively modulate the pathway in specific cell types, for instance, enhancing the protective CX3CR1 signaling in Kupffer cells or monocytes without fueling pro-tumorigenic or pro-inflammatory responses in other compartments (6, 12). Promisingly, preclinical studies provide a strong rationale for several interventional paradigms: (1) direct pathway blockade using neutralizing antibodies or small-molecule receptor antagonists; (2) upstream regulation, such as employing microRNA-based therapies (e.g., miR-561-5p) to control CX3CL1 expression (11); and (3) modulation of downstream effectors, exemplified by using molecules like PD1 to suppress the pathogenic NF-κB-mediated arm of the axis (27).

Future drug development must prioritize overcoming these challenges by exploiting the most promising scientific opportunities. The immediate priority is to advance the most validated targeting strategies, such as CX3CL1-neutralizing antibodies or small molecule CX3CR1 antagonists, into early-phase clinical trials for specific indications like HCC, where the pro-tumorigenic role is well-established. A highly promising direction is developing rational combination therapies, such as integrating CX3CL1/CX3CR1 axis inhibition (to deplete immunosuppressive MDSCs) with immune checkpoint blockade to achieve synergistic anti-tumor efficacy (12, 39). For complex metabolic diseases like NASH, research should focus on identifying the precise signals that determine the axis’s switch from protective to pathogenic, which could unlock the potential for conditional or tissue-specific agonism (42). Ultimately, success will depend on the development of sophisticated delivery systems that can target modulatory agents to specific hepatic cell populations, thereby maximizing therapeutic benefit while minimizing off-target effects.

## 8 Translational challenges and future directions in CX3CL1/CX3CR1 research in liver diseases

The conspicuous absence of CX3CL1/CX3CR1-targeting agents in clinical trials for liver diseases is not due to a lack of mechanistic rationale but stems from profound translational challenges. First, the pleiotropic physiology of the axis presents a major pharmacological hurdle. Its critical roles in neuronal communication and microglial homeostasis mean that systemic inhibition, particularly with brain-penetrant small molecules, carries a tangible risk of CNS side-effects, such as exacerbating neuroinflammation (35). Second, achieving cell- and context-specificity is a formidable task. The axis mediates opposing functions even within the liver: it protects against fibrosis by regulating monocyte fate (6, 28) and maintains intestinal barrier integrity (43), yet drives pathogenesis by recruiting immunosuppressive MDSCs in HCC (12, 39). A therapeutic agent must precisely inhibit the pathogenic arm without disrupting these protective functions, a selectivity barrier not yet overcome. Third, modality-specific limitations exist. Neutralizing antibodies offer high specificity but may have poor tissue penetration and cannot distinguish between pathogenic and homeostatic ligand pools. Small-molecule receptor antagonists face the aforementioned systemic safety concerns. While elegant, miRNA-based strategies (e.g., targeting miR-561-5p (11)) or pro-resolving mediators (e.g., Protectin D1 (27)) are in early development, challenged by delivery and stability issues.

Future research must focus on three interconnected pillars: resolving the functional paradox of this axis, developing precise interventional strategies, and building a bridge to clinical translation. The foremost priority is a deep mechanistic investigation, moving beyond phenotypic associations to systematically decipher the molecular switches and cell-specific signaling pathways that determine its functional shift towards protection” or damage, ” which forms the theoretical foundation for precise targeting. Building on this, therapeutic development should adhere to a context-specific” principle: in clear pathological contexts like liver cancer, exploring synergistic combinations of axis inhibitors (e.g., targeting MDSC recruitment) with existing immunotherapies; for diseases like NASH where the axis plays a protective role, the focus should be on designing localized or conditionally activated intelligent delivery systems. Ultimately, the success of all translational efforts hinges on the discovery and validation of predictive biomarkers, capable of accurately identifying patient subsets whose disease progression is genuinely driven by this axis, thereby achieving a fundamental leap from broad association” to guiding clinical application” in precision therapy.

## 9 Discussion

The evidence synthesized in this review compellingly argues against a simplistic, binary interpretation of the CX3CL1/CX3CR1 axis in liver health and disease. Its defining characteristic is not a fixed pro- or anti-disease function, but a profound context-dependent duality. This very complexity, however, is the key to its therapeutic promise. The central challenge for the field is no longer to catalog this paradox but to systematically decode it, transforming an apparent biological contradiction into a blueprint for precision intervention.

To achieve this, we propose a focused, tripartite framework to guide future research and translation. The first pillar is mechanistic decoding, which necessitates a shift from disease-specific observations to comparative studies that dissect the precise variables—such as the cellular source of CX3CL1, the phenotype of recruited immune cells (16, 17), and crosstalk with dominant signaling nodes—that flip the functional switch” between protective and pathogenic outcomes.

The second pillar is therapeutic precision, which demands that intervention strategies be as context-aware as the biology itself. This means developing targeted antagonists for well-defined pathogenic circuits (e.g., in HCC) (13, 41), while exploring conditional or localized strategies (e.g., tissue-restricted delivery, indirect pathway modulation) (33)for contexts where the axis exerts protective effects, thereby avoiding the pitfalls of systemic manipulation.

The final, indispensable pillar is translational bridging. The current lack of clinical trials stems from valid safety concerns and the absence of tools to identify patients who would truly benefit. Therefore, parallel development of sophisticated predictive biomarkers—moving beyond simple ligand measurement to assess the functional state of the axis—and a deeper understanding of its modulators (44)—is critical for patient stratification and de-risking clinical development.

To operationalize this contextual framework, it is essential to map it onto the disease continuum itself. Reviewing the axis’s actions through the lens of the classic chronic inflammation-fibrosis-cancer progression provides crucial pathophysiological insight. The evidence confirms that the CX3CL1/CX3CR1 axis is not merely a bystander but an active participant at each stage. Its function, however, is not linearly pro- or anti-disease. Instead, it can be re-wired by the evolving tissue microenvironment—from the initial inflammatory insult, through the remodeling extracellular matrix during fibrosis, to the immunosuppressive and pro-angiogenic niche of the tumor. For instance, its protective role in regulating monocyte infiltration during fibrosis (6, 28) can be subverted in the established tumor, where similar recruitment mechanisms bolster immunosuppressive MDSC populations (12, 39). This plasticity underscores why therapeutic targeting must be context-specific, informed by the dominant disease stage and the resultant cellular and signaling networks in which the axis is embedded.

In conclusion, the future of targeting the CX3CL1/CX3CR1 axis lies in embracing its contextual logicacross the disease timeline. By integrating deep mechanistic understanding with intelligent therapeutic design and robust biomarker science, this pathway can evolve from a fascinating biological puzzle into a cornerstone of personalized medicine in hepatology.
